# Grammatical devices of stance in written academic English

**DOI:** 10.1016/j.heliyon.2021.e08463

**Published:** 2021-11-27

**Authors:** Sharif Alghazo, Mohd Nour Al Salem, Imran Alrashdan, Ghaleb Rabab'ah

**Affiliations:** a University of Jordan, Jordan; b University of Sharjah, United Arab Emirates

**Keywords:** Stance, Academic English, Research article abstracts, Applied linguistics, Literature

## Abstract

Stance is a feature of academic writing that refers to how writers interact and engage with their readers by means of linguistic devices. This study focuses on the grammatical devices—and semantic distinctions thereof—that are employed by academic writers of English to express stance in research article abstracts in the areas of applied linguistics (AL) and literature (L). To this end, a corpus of 120 research article abstracts (60 in the area of AL and another 60 in that of L) was built and analysed using SPSS and following Biber et al.’s (1999) framework of grammatical devices of stance. The abstracts were extracted from high-quality journals in the respective areas: *Applied Linguistics* and *English: Journal of the English Association*. Both are ISI journals and published by Oxford Academic Publishing. A mixed-method approach, applying quantitative and qualitative measures, was adopted to answer the two questions: How is stance grammatically expressed in AL research article abstracts and L research article abstracts, and How is the expression of stance in AL research article abstracts similar to/different from that in L ones? The findings are construed in light of theories of academic discourse and English for Academic Purposes (EAP). The results reveal that there are important similarities and differences in the extent to which and the means through which stance is expressed in AL research article abstracts and L research article abstracts. In particular, the findings show that both AL and L abstracts were similar in the most frequently used stance marker which is the stance complement clause. However, they were different in the frequency of use of other devices. The study provides insights into the ways academic writers express stance in various fields which better our ability to write research article abstracts.

## 1 Introduction

Academic writing is a mode of communication that involves an interaction between writers and readers by means of linguistic devices (Jin, 2015). As Hyland (2005) notes, academic writers seek to establish for a relationship with their readers by actively interacting with them. This kind of interaction is technically referred to as stance which is defined by Strauss and Feiz (2014) as “the speaker's or writer's feeling, attitude, perspective, or position as enacted in discourse” (p. 103). Stance taking refers to one taking a position to express her/his attitudes, opinions or ideologies towards something or someone. Jiang and Hyland (2015) define stance-taking as “the means by which academics take ownership of their work: making epistemic and evaluative judgement regarding entities, attributes and the relations between material to persuade readers of their right to speak with authority and to establish their reputations” (p. 20). Pho (2013) adds that stance is “the writer's *identity* as well as the writer's expression of attitudes, feelings, or judgments” (p. 3; italics added). This identity is constructed and communicated to readers by means of a variety of lexical and grammatical devices that are differently used according to register.

Register is a discourse-related term that “refers to the sets of grammatical, lexical, and prosodic features of discourse within genres that together signal or index membership within a specific group …. As such, *registers* also index ideologies and *identities*” (Strauss and Feiz, 2014, p. 72; the first italics is in original, and the second is added). For example, Biber (2006), in his investigation of spoken and written university registers, showed clear differences in the expression of stance based on register. In keeping with Strauss and Feiz's (2014) definition of register above, Biber (2012) argues that the linguistic devices—lexical and grammatical—and their semantic distinctions represent a major component of the description of register. The other two components are the situational context and its relationship with the linguistic devices.

Research in areas of academic discourse and genre analysis has focused on the linguistic devices (lexico-grammatical features) that writers use to interact with readers by expressing their attitudes and feelings (e.g., Gray and Biber, 2012, 2014; Hyland, 2005; Jalali, 2017, among many others). In their seminal work, Biber et al. (1999) assert that lexical stance marking is limited in that it encompasses only one proposition and does not “provide attitudinal and evaluative frame for other propositions” (p. 966). In addition, studies have investigated comparatively the expression of stance in various registers (e.g., Biber, 2006) and languages (e.g., Biber, et al., 2006). However, the expression of stance in sub-disciplines has not been given enough attention from researchers. Thus, this study plugs this gap and explores the grammatical devices of stance in AL research article abstracts and L ones by seeking answers to the following research questions:

1. How is stance grammatically expressed in applied linguistics research article abstracts and literature research article abstracts?
2. What are the differences and/or similarities (if any) in the use of grammatical devices to express stance in both types of abstracts?

## 2 Literature review

The literature on stance marking in academic writing varied in its focus and scope. Some studies, for example, were monolingual and monocultural in the sense that stance marking was investigated within the same language (e.g., Biber, 2006). Other studies were cross-cultural. The literature on stance marking abounds with studies whose aim is to compare the expression of stance in academic writing across languages and cultures (e.g., Alghazo et al., 2021; Yu, 2019; Šinkūnienė, 2018). In monolingual and monocultural research, many studies focused on specific grammatical devices and their distribution across disciplines (e.g., Hyland and Tse, 2005). Other studies were more holistic in nature encompassing a wide range of stance marking (e.g., Hyland, 2005). This study falls within the latter type and aims to explore stance marking in English academic writing in the fields of applied linguistics and literature.

### 2.1 Monocultural studies

As mentioned above, some of the monolingual studies aimed to explore the grammatical devices that writers use to express stance; one of these is the noun complement construction (e.g., the assumption that …). Jiang and Hyland (2015) explored the use (by measures of frequencies), forms and functions of the noun complement construction. The data were taken from a corpus (1.7 million words) comprising 160 research articles from eight disciplines. The concordance software AntConc was employed to spot the following structures: The N *that*, N *to-infinitive,* and N *of-preposition* structures. In addition, the researchers conducted a manual reading of the articles to ensure that all noun complement clauses have been identified. The focus was on the semantic rather than the functional characteristics of the noun complement clauses. The results show that writers’ stance towards attributes of entities is the most used, with 25% of all stance nouns. The findings also reveal that stance nouns which refer to objects and relations are the least used. It has also been revealed that noun complements are used more in the soft fields such as applied linguistics, marketing, and sociology than in the hard ones like engineering. All in all, the study emphasize that stance is not only a lexical feature in discourse but also a grammatical one.

In an earlier study, Hyland and Tse (2005) investigated the use of evaluative *that* constructions as one of the interpersonal features academic writers use to express stance in their writings. The data included 240 abstracts from different disciplines which were classified into categories and subcategories. The results about the frequency of use of *that* show a significant difference between the abstracts of articles in the different disciplines covered in the study. *That* constructions were found to be more frequent in computer sciences and business studies, with 8.5 and 8.0 that word per 1000 words collected in both sciences respectively. Conversely, *that* constructions were the least frequent in electronic engineering with 2.1 that word per 1000 words. The findings demonstrated that academic writers make use of *that*-clause in their abstracts to give comments and to validate their conclusions and claims.

In another study, Jalali (2017) investigated the use of a particular group of lexical bundles in the expression of stance. The data were taken from three corpora: research articles, doctoral dissertations, and master's theses, all in the field of applied linguistics. The use of these bundles was compared across the three corpora. More specifically, the aim was to compare between published writers and students in terms of styles of writing. The results reveal the use of 17 different bundles identified in the corpus of research articles. *It bundles* such as *it is important to*, *it should be noted*, *it is necessary to* and *it is not clear* are used in all three corpora. *It bundles* such as *it is clear that*, *it is interesting to*, *it is hoped that,* and *it is suggested that* were not employed frequently in the postgraduate writings. The use of the *it bundles* was similar in doctoral dissertations and master's theses as the difference was not significant. There were certain differences, however, in the average of use of *it bundles* such as *it should be noted, it is difficult to* which were identified more in the master's theses compared to the doctoral dissertations. It has been found that skilled writers in the field of applied linguistics resort to the use of such bundles to express their position, stance, and interpersonal meanings. The functional analysis reveals that the use of these lexical bundles in the research articles was more significant compared to the doctoral dissertations and master's theses. This may be due to the degree of confidence that skilled writers show in research articles which gives them more space and freedom to take stance and express themselves.

Some monolingual studies concentrated on how the use of stance markers changes over time. For example, Hyland and Jiang (2017) explored how the use of ‘Evaluative that’ has/not changed over the past 50 years. To this end, three corpora of research articles from five journals in four disciplines (viz., applied linguistics, sociology, biology, and electronic engineering) spaced at three periods: 1965, 1985 and 2015 were constructed. Overall, the corpus comprised 360 papers and a total of 2.2 million words. The results show that there has been a substantial increase in the use of *evaluative that* constructions over the past 50 years. Decreases were recorded in biology and applied linguistics with the sharpest decrease after 1985 seen namely in sociology and electrical engineering. This was attributed to a ‘stylistic shift’ in academic claim-making. Despite the decline of its popularity in the past 50 years across all the four disciplines, *evaluative that* constructions remain a significant rhetorical option for authors to express stance. The decline of this structure by about 20% since 1965 entails that there are alternative epistemic resources available to authors (single modal items) which allow more succinct expressions and a more compact style of argument. Such findings call for more studies on diachronic changes of other stance markers used in English academic writing.

A similar study was conducted by Hyland and Jiang (2018) on changes to the use of various metadiscourse devices. The study aimed to explore changes in metadiscourse using corpus analysis. Three corpora were created consisting of 30 articles from five journals in four disciplines (applied linguistics, sociology, electrical engineering, and biology) at three periods over the past 50 years: 1965, 1985 and 2015. The data were analysed using AntConc and focused on explicit textual devices analysed based on Hyland's (2004, 2005) model. The results reveal that there was a statistically significant increase in the use of interactive features in all fields since 1965, viz., endophorics, code glosses and evidentials which nearly doubled. This was attributed to writers seeking to enhance the cohesion and explicitness of their arguments. Interactional resources, on the other hand, have seen a 6.5% decrease with devices in all categories. Boosters and attitude markers have shown the steepest decline over the last 50 years. Thus, writers are using more features to guide readers through more explicitly cohesive texts and fewer features to take a personal stance and engage directly with readers.

In a comparative monolingual study that investigated both disciplinary differences and academic levels of writers, Akinci (2016) explored differences in stance marking devices in the writings of student writers and academics. To this end, the researcher analysed a corpus of 39 research articles using Hyland's (2005) framework of metadiscourse to find differences according to disciplines—in this case Civil Engineering and Applied Linguistics—and according to level of writers, i.e., student writers and expert academics. The findings reveal that more stance markers were used in student writers' articles than those in the academics' articles. In addition, the results show that there exist some cross-disciplinary differences in terms of stance markers. In particular, the study found that Applied Linguistics research articles included more stance marking devices than did the Civil Engineering articles. The results also demonstrate that student writing featured more stance, most notably in the use of self-mentions. Such findings have insights for both students and teachers on how to address stance making in writing courses.

### 2.2 Cross-cultural studies

Cross-culturally, fewer studies were conducted to explore differences across languages in how stance is expressed. Of these studies is that by Yu (2019) who examined cross-cultural differences in how academic writers express authorial stance by analysing corpora of published research articles in English and Korean. To achieve this objective, the researcher built two corpora of Applied Linguistics research articles in the two languages. Each corpus contained 50 articles. A mixed-method approach was used in the analysis of stance marking devices in the two corpora. The results showed that there are differences in the ways through which writers express their stance towards propositions in English and Korean. These differences were attributed to cultural differences that are reflected in writing—which is essentially a reflective task. The study provided repercussions for which to address these differences in academic and research writing courses.

Space does not permit a comprehensive review of cross-cultural studies on the use of stance marking devices. This endeavour is beyond the scope of this study which exclusively focuses on English. However, and based on the foregoing, we notice that despite the plethora of cross-disciplinary research in relation to stance marking in English which compares highly divergent disciplines, very few studies look at differences between related genres or sub-disciplines. To fill this gap, this study explores how stance is expressed by writers of research article abstracts in the two related areas of Applied Linguistics and English Literature. In addition, most studies are limited in terms of the scope of the linguistic devices investigated, with the reviewed studies mostly examining the use of only one device at a time. However, this study is more encompassing and comprehensive. Finally, most studies followed Hyland's and others' models which criticised for being limited in scope. Biber et al. (1999) is more comprehensive in its coverage of the linguistic devices of stance.

## 3 Methodology

### 3.1 Corpus

The researchers designed a corpus that consists of 120 research article abstracts (60 in the area of AL and 60 in that of L). The abstracts were extracted from research articles published in two highly-indexed journals in the respective fields—*Applied Linguistics* and *English: Journal of the English Association*. Both are ISI journals and published by Oxford Academic Publishing. The research articles were published between 2014 and 2020. Nativeness to English was not an important variable in this study because it is the language used, rather than the writer, that is being investigated. The whole corpus resulted in 25,560 words (12,430 words in AL and 13,130 in L). The study was designed to allow for a comparison between AL research article abstracts and L ones in terms of the grammatical devices and their semantic distinctions that writers use to express stance.

### 3.2 Data coding and data analysis

As for the analysis, Biber et al.’s (1999) framework of the grammatical marking of stance was adopted to analyse grammatical stance markers in the two corpora. Biber et al.’s (1999, pp. 969–970) model of stance marking comprises an exhaustive list of linguistic devices that is categorised under five main classes: stance adverbials, stance complement clauses, modals and semi-modals, stance noun + prepositional phrase, and premodifying stance adverb (stance adverb + adjective or noun phrase). Each type is manifested in several grammatical constructions that function as stance marking. Table 1 below shows Biber et al.’s (1999) framework of grammatical stance marking and examples on each marker from Biber et al. (1999).

**Table 1:** Biber et al.'s (1999) framework of grammatical stance marking.

Grammatical Stance Marker	Grammatical Constructions	Examples
**A. Stance Adverbials**	1. Single adverbs and adverb phrases	*Unfortunately,* we cannot do anything about it
	2. Hedges	He's *kind of* talked himself into it.
	3. Prepositional phrases	*In actual fact* only a fraction of this number actually occurs.
	4. Adverbial clauses	*As one might expect*, Gauss didn't collaborate much with others.
	5. Comment clauses	You just have to try and accept it, *I guess*.
**B. Stance Complement Clauses**	1. Controlled by a verb	I just *hope* that I've plugged it in properly.
	2. Controlled by an adjective	I'm *very happy* that we're going to Sara's.
	3. Extraposed structures	It's *amazing* that judges can get away with outrageous statements.
	4. Controlled by a noun	The *fact* that he will get away with attacking my daughter is obscene.
**C. Modals and Semi-modals**	Might, Has to, Must, May	I *might* be up before you go. She *has to* go to a special school.
**D. Stance Noun + Prepositional Phrase**	Possibility, Fear	They deny the *possibility* of a death wish lurking amidst the gardens of lust.
**E. Premodifying Stance Adverb (Stance Adverb + Adjective or Noun Phrase**	So, Really, About	I'm *so* happy for you. I'm *really* happy for you.

By applying Biber et al.’s (1999) framework, the researchers used a mixed-method approach to analyse the data, employing both quantitative and qualitative measures. The former method resulted in statistical presentation of the frequencies of occurrence of each grammatical device in the two corpora. The latter was necessary to apprise readers of the way/s each device was used to express stance and to provide them with authentic examples that show the similarities and differences between AL research article abstracts and L ones in the use of grammatical stance marking. In order to analyse the data, the researchers surveyed Biber et al.’s (1999) source for all possible linguistic devices that are used to express stance and made a list of these devices. In addition, each researcher perused the abstracts so as to present a precise count of the grammatical devices in each abstract. In order to validate the individual analysis of the abstracts, a collective session of negotiation among the researchers resulted in a more robust analysis of the various stance markers.

## 4 Results

This section presents the findings of the study. As noted earlier, there were two methods of data analysis: quantitative and qualitative. The first part of this section is devoted to the quantitative analysis of the grammatical devices that were found in the two corpora highlighting similarities and/or differences (if any) between the AL research article abstracts and L ones. The second part presents examples that show the semantic distinctions of stance in both corpora highlighting disciplinary features of stance in academic research writing.

### 4.1 Quantitative findings

The data analysis shows that there exists a variation in the use of various stance-marking devices in the two corpora. The following table shows frequencies and percentages of occurrences of grammatical devices of stance in the AL and L abstracts corpora. The percentages are counted per 1000 words. In addition, the table shows the results of the Chi^2^ test and the significance value of the comparison.

Table 2 above shows that the most common type of grammatical stance marking in the AL research article abstracts was *stance complement clauses,* with 216 occurrences. Jiang and Hyland (2015) argue that the noun complement structure is “relatively overlooked” (p. 1) a means of marking stance which plays a role in creating cohesion and in conveying the attitudes of the writer. Within this type, *clauses controlled by a verb* were the most used, with a frequency of 174 occurrences (1.40%). *Adjective-controlled clauses* came second, with 24 occurrences and *extraposed structures* third, with 18 occurrences. There were no *noun-controlled clauses* in this corpus. The second most common type of grammatical stance marking in AL research article abstracts was *modals and semi-modals,* with 60 occurrences. This was followed by *stance noun + prepositional phrase,* with 48 occurrences. *Stance adverbials* came next with 30 occurrences. Most frequent stance adverbials were *single adverbs and adverb phrases* (24 times) and some were *hedges* (6 occurrences). There were no instances of other types of *stance adverbials* such as *prepositional phrases, adverbial clauses,* and *comment clauses*. Finally, there were 12 instances of *premodifying stance adverbs.*

**Table 2:** Frequencies and percentages of stance markers in the two corpora.

Grammatical Stance Marker	Grammatical Constructions	AL		L		Chi^2^	Sig.
		Frequency (12,430)	Percentage (1000)∗	Frequency (13,130)	Percentage (1000)∗		
**A. Stance Adverbials**	1. Single adverbs and adverb phrases	24	0.002	12	0.0009	4.000	0.046∗
	2. Hedges	6	0.00048	0	0.00%	-	-
	3. Prepositional phrases	0	0.00%	12	0.00093	-	-
	4. Adverbial clauses	0	0.00%	42	0.0032	-	-
	5. Comment clauses	0	0.00%	6	0.0004	-	-
	**Total**	**30**	**0.0024%**	**72**	**0.0055%**	**17.294**	**0.00**∗
**B. Stance Complement Clauses**	1. Controlled by a verb	174	0.014	132	0.010	5.765	0.016∗
	2. Controlled by an adjective	24	0.002	30	0.0022	0.667	0.414
	3. Extraposed structures	18	0.0014	18	0.0014	0.111	0.739
	4. Controlled by a noun	0	0.00	6	0.0004	-	-
	**Total**	**216**	**1.74%**	**186**	**1.42%**	**1.180**	**0.277**
**C. Modals and Semi-modals**	Might, Has to, Must, May	60	0.0048	54	0.0041	0.316	0.574
**D. Stance Noun + Prepositional Phrase**	Possibility, Fear	48	0.0038	84	0.0064	5.541	0.019∗
**E. Premodifying Stance Adverb (Stance Adverb + Adjective or Noun Phrase)**	So, Really, About	12	0.00096	6	0.0004	2.000	0.157
*Total*		*366*	*0.029*	*402*	*0.030*	*1.688*	*0.194*

∗ Percentages are counted per 1000 words.

As for the L abstracts, Table 2 above demonstrates that the most common type of grammatical stance marking in was *stance complement clauses,* with a frequency of 186 occurrences. Within this type, *clauses controlled by a verb* were the most used, with a frequency of 132 occurrences (1.40%). *Adjective-controlled clauses* came second, with 30 occurrences and *extraposed structures* third, with 18 occurrences. There were six occurrences of *noun-controlled clauses* in this corpus. The table also shows that *stance noun + prepositional phrase* was the second most frequent stance device (84 occurrences) in the L corpus. *Stance adverbials* scored third in this corpus in terms of frequency of occurrence with 72 times. Most stance adverbials were *adverbial clauses* (42 times; *single adverbs and adverb phrases* and *prepositional phrases* occurred 12 times each. There were six instances of *comment clauses*, but none of *hedges*. *Modals and semi-modals* came fourth, with 54 occurrences. The least frequently used stance marker was *premodifying stance adverb (stance adverb + adjective or noun phrase* with only 6 occurrences.

Table 2 above shows that the grammatical stance devices in the AL abstracts corpus were (12.430), and the grammatical stance devices in the L abstracts corpus were (13.130). The single adverbs and adverb phrases were (24) in the AL abstracts with a percentage of (0.002/1000 words). On the other hand, the single adverbs and adverb phrases were (12) in the L abstracts corpus, with a percentage of (0.0009/1000 words). The (Chi^2^) value was (4.000), its significance is at (0.05), and the variance was in favour of the AL abstracts corpus. The hedges were only found in the AL abstracts corpus, with a percentage of (0.00048/1000 words). In addition, the results reveal that there were no prepositional phrases, adverbial clauses and comment clauses in the AL abstracts corpus. However, the L abstracts corpus contained prepositional phrases, with a frequency of (12) and a percentage of (0.00093/1000 words) and adverbial clauses with a frequency of 42 and a percentage of (0.0032/1000 words). The comment clauses were (6) with a percentage of (0.0004/1000 words) in the L abstracts corpus. The total number of stance adverbials in the AL abstracts corpus was (30), with a percentage of (0.0024/1000 words) and that in the L abstracts corpus was (72), with a percentage of (0.0055/1000 words). The results show that there were statistically significant differences between AL and L abstracts corpora (Chi^2^ = 17.294) which is significant at a level of 0.05 and the variance is in favour of L abstracts corpus.

As for the stance complement clauses, the results show that the clauses which are controlled by a verb in the AL abstracts corpus were (174), with a percentage of (0.014/1000 words) while the occurrences of the same grammatical construction in the L abstracts corpus were (132), with a percentage of (0.010). The findings reveal that there are statistically significant differences between the AL and L abstracts corpora (Chi^2^ = 5.765), and its significance is at a level of (0.05) which implies that the variance is in favour of the AL abstracts corpus. The results also show there are no statistically significant differences between the AL and L abstracts corpora in relation to the stance complement clauses which are controlled by an adjective because the frequency for the AL abstracts corpus is (24) = (0.002/1000 words) and for the L abstracts corpus is (30) = (0.0022/1000 words); the Chi^2^ = 0.667 and this is not significant at the level of (0.05). The extraposed structures were equal between the AL and L abstracts corpora, with a frequency of (18) for each, and a percentage of (0.0014/1000); the Chi^2^ value was (0.111) which is not significant at the level of (0.05). The stance complement clauses which are controlled by a noun were absent in the AL abstracts corpus, but there were (6) occurrences in the L abstracts corpus, with a percentage of (0.0004/1000 words).

The third category of stance markers is modals and semi-modals. The results presented in Table 2 above demonstrate that there is a frequency of (60) = (0.0048/1000 words) in the AL abstracts corpus and a frequency of (54), with a percentage of (0.0041/1000 words) in the L abstracts corpus. The results show that there were no statistically significant differences between the AL and L abstracts corpora (Chi^2^ = 0.316), and this is not a significant difference at the level of (0.05).

The fourth category is the stance noun + prepositional phrase. Here, the AL abstracts contain (48) occurrences, with a percentage of (0.0038/1000 words), and the L abstracts contain (84) occurrences, with a percentage of (0.0064/1000 words). The Chi^2^ result is 5.541 which is significant at the level of (0.05). This indicates that there are significant differences between the two corpora, and that the variance is in favour of the L abstracts corpus.

As for the premodifying stance adverbs (stance adverb + adjective or noun phrase), the results show that the AL abstracts corpus includes a frequency of (12), with a percentage of (0.00096/1000 words), and the L abstracts corpus has a frequency of (6), with a percentage of (0.0004/1000 words). The Chi^2^ value is (2.000) which is not significant at the level of (0.05).

Finally, the total number of grammatical stance devices in the AL abstracts corpus was (366) words out of (12430), with percent of (0.029/1000 words) whereas the grammatical stance devices in the L abstracts corpus were (402) words out of (13,130), with a percentage of (0.030). The Chi^2^ value is (1.688) which is not significant at the level of (0.05).

If we compare the two corpora in terms of grammatical stance marking, we notice that both the AL and L abstracts share a similarity in that stance complement clauses were the most frequent. This emphasises the argument put forth by Jiang and Hyland (2015) that stance complement clauses are devices employed by writers to tie segments of the text in a harmonious manner. However, the analysis shows that more occurrences of these markers were spotted in the AL corpus than were found in the L one. In addition, while there were six occurrences of *noun-controlled clauses* in the L corpus, there was no instance of this marker in the AL one. Finally, while *stance noun + prepositional phrase* came second on the list of most frequent devices in the L corpus, *modals and semi-modals* occupied this position in terms of frequency in the AL corpus with *stance noun + prepositional phrase* being third on the list.

### 4.2 Qualitative findings

This sub-section is devoted to presenting a qualitative analysis of the data. It illustrates through authentic examples how each existing grammatical stance marker is used in the two corpora of AL and L research article abstracts. As found in the quantitative analysis above, stance complement clauses were the most frequent stance marker in the AL corpus. This marker, as Biber et al. (1999) note, is realised in four grammatical constructions. However, only three existed in the AL corpus. Examples on each existing construction are presented below.

1. Stance Complement Clause

As mentioned above, stance complement clauses can be controlled by a verb, an adjective, or a noun. They can also be extraposed structures. In this corpus, no instances of noun-complement clauses were found. The other three constructions (i.e., controlled by a verb, controlled by an adjective and extraposed structures) were found as in the following examples:

a. These findings *suggest* that ways of navigating the body in the classroom space can index pedagogical concerns….
b. By drawing on critical language policy, it *appears* that policy may be so ethnonationalist that it has caused disassociation….
c. Results indicate that it is *possible* to identify particular features of English speech varieties that are most likely to lead to a breakdown in communication.
d. It is *now 20 years* since ‘social remittances’ was taken up to capture the notion that … more tangible circulation of money.

As can be seen, the first two examples above show stance complement clauses controlled by the two verbs *suggest* and *appear*, respectively and, in (b), by the evaluative adjective ‘ethnonationalist’. The third and fourth examples demonstrate how the stance complement clauses ‘to identify … communication’ and ‘since … money’ are extraposed structures that are used to express stance.

2. Modals and Semi-modals

The second most common type of grammatical stance marking in AL research article abstracts was *modals and semi-modals,* with 60 occurrences. Examples are the following:

e. From, respectively, a distinctive collexeme … and behavioral profile analysis … will emerge that beyond expressions of joint attention, children's ToM ability progressively underpins ….
f. However, SEM also suggested that the recognition and recall masteries of any particular word knowledge component must be seen as separate constructs.
g. This article shows, with Malaysia as a case study, that an ethnonationalist language policy need not have disempowering consequences for minorities.

The examples above show that modals as stance markers have different semantic distinctions. In (e), the modal *will* is used to express prediction or volition. In (f), *must* is used to express obligation, and in (g), the semi-modal *need not* is used “as a frame for the interpretation of the propositional information” (Biber et al., 1999, p. 971).

3. Stance Noun + Prepositional Phrase

As noted above, stance noun + prepositional phrase markers scored third in the AL corpus in terms of frequency, with 48 occurrences. Biber et al. (1999) warn that although stance noun + prepositional phrase markers “have two distinct components, … it is not always clear that the prepositional phrase actually presents a ‘preposition’” (p. 970). Therefore, care must be taken when analysing such markers. Below are two examples from the corpus.

h. The findings confirm the applicability of CDST approaches to L2 oral development and carry valuable implications for CDST theory development and oral language teaching.
i. Learning a visual language gives hearing mothers the possibility of participating in their deaf children's culture.

We notice that, in the two examples above, the stance nouns *applicability* and *possibility* were both followed by prepositional phrases that present propositions. Such uses are common in academic writing, as noted by Biber et al. (1999).

4. Phrase Stance Adverbials

The quantitative analysis presented above shows that phrase stance adverbials came fourth in terms of frequency, with 30 occurrences. It should be recollected that most stance adverbials were *single adverbs and adverb phrases* (24 times) and some were *hedges* (6 occurrences). There were no instances of other types of *stance adverbials* such as *prepositional phrases, adverbial clauses,* and *comment clauses*. Below are some examples that show their occurrences.

j. Unlike continuous whole-class ‘plenary’ interaction, independent task work involves incipient teacher-student talk, as the teacher typically ‘makes round’ to engage … with students.
k. Results indicate that it is possible to identify particular features of English speech varieties that are most likely to lead to a breakdown in communication.

The two examples (j & k) demonstrate that phrase stance adverbials express different semantic distinctions. For example, *typically* in (j) shows an epistemic condition (see Biber et al., 1999) on the proposition, that of a limitation. *Most likely* in (k) expresses an epistemic condition of certainty on the proposition. As Biber et al. (1999) argue, phrase stance adverbials are usually used to “convey the speaker/writer's assessment of the proposition in the clause” (p. 549).

5. Premodifying Stance Adverbs

The least frequently used stance marker in the AL corpus was premodifying stance adverbs, with 12 instances. Two examples are presented below in which *so* and *very* are used.

l. By drawing on critical language policy, it appears that policy may be so ethnonationalist that it has caused disassociation.
m. However, research systematically investigating the threshold of intelligibility has been very limited.

If we turn to the L corpus, we find, as noted above, some similarities to and differences from the ways in which these stance markers are used in the AL research article abstracts—although the difference in the total number of stance markers in the two sets of data was not significant (29.45/1000w for the AL corpus and 30.6/1000w for the L one, see Table 2 above). For example, stance complement clauses were—similar to the AL set—the most frequent in the L corpus. However, there was some variegation in the use of structural manifestations of this marker. That is, all four constructions of stance complement clauses were found in this corpus, as follows:

1. Stance Complement Clause

Stance complement clauses were the most used in the L corpus. Within this type, *clauses controlled by a verb* were the most used, with a frequency of 132 occurrences (1.40%). *Adjective-controlled clauses* came second, with 30 occurrences and *extraposed structures* came third, with 18 occurrences. There were six occurrences of *noun-controlled clauses* in this corpus, as shown in the following examples:

a) I will also *argue* that it has been brought surprisingly to the fore in two recent experimental texts, Eimear McBride's *The Lesser Bohemians* and Nicola Barker's *H(A)PPY.*
b) In particular, it *suggests* that there are fruitful connections to be made between modern posthumanist theoretical approaches, and the post-humanism of Higgins's approach to exemplary history, whereby his admonitory text *appears* to abandon its premise of human primacy and perfectability in response to the perceived failure of Elizabethan advice literature to effect political change.
c) For Nashe, … it was *much more important* to concentrate on the locality of England itself.
d) Identifying the importance of these techniques to Wordsworthian elegy, Shelley's sonnet ‘To Wordsworth’ shows him inheriting Wordsworth's *belief* that any elegy must negotiate between ‘common woes’ and individual feeling.

In the examples above (a-d), we find how stance complement clauses are used in the L corpus. In (a & b), the clauses are controlled by the verbs *argue* and *suggest*, respectively. In (c), there is an example on an extraposed structure in which the complement clause is extraposed by the subject *it*. In (d), the complement clause is led by the noun *belief*.

2. Stance Noun + Prepositional

The quantitative analysis above has shown that *stance noun + prepositional phrase* was the second most frequent stance device (84 occurrences) in the L corpus. Some examples are presented here to show how this device was used.

e) With different emphases, a number of critics have attended to the significance of silence in Wordsworth's poetry.
f) Through this, Wordsworth questions the possibility of the elegist and the reader experiencing a unified response to loss.

In the two examples above, the stance nouns *significance* and *possibility* were both followed by prepositional phrases that present propositions.

3. Phrase Stance Adverbials

*Phrase stance adverbials* scored third in the L corpus in terms of frequency of occurrence, with 72 times. As shown in the previous section, most stance adverbials were *adverbial clauses* (42 times); *single adverbs and adverb phrases* and *prepositional phrases* occurred 12 times each. There were six instances of *comment clauses*, but none of *hedges*. This use is illustrated by the following example:

g) I will also argue that it has been brought surprisingly to the fore in two recent experimental texts …. At first glance, the marriage of experimentalism and happiness may appear odd; as Sianne Ngai observes, the avant-garde ‘is conventionally imagined as sharp and pointy, as hard- or cutting-edge’, and Rachel Greenwald Smith has delineated a supposed tension between affect and postmodernism.
h) For his contemporary reviewers*,* who set the tone of his reception, he never quite escaped his association with Greenwich Village post-Decadence.
i) This subject, so it is argued – the place of scholarship undertaken on an unaffiliated or independent basis in the world of English studies – is a topic worthy of sustained attention.

The first example shows two instances of phrase stance adverbials. The first is an instance of a single adverb (surprisingly), and the second is an adverbial clause. In (h), a prepositional phrase (for his contemporary reviewers) is used to express the stance of writer while, in (i), the comment clause *so it is argued* is used.

4. Modals and Semi-modals

Analysis of the data shows that modals and semi-modals occurred 54 times in the L corpus. Below are some examples that illustrate their use by the writers of the selected abstracts.

j) Finally, this article demonstrates how patterns established in his immediate reception are reproduced in later criticism—and may even explain his relative critical neglect.
k) Virginia Woolf's *The Waves* is a difficult novel to comprehend rationally, and yet it is also a very moving novel that can take the reader from ecstasy to despair.

Here, the modal *may* in (j) is used to express the meaning of possibility, and the modal *can* in (k) to express the ability function, stances taken by the writers towards the proposition which follow the auxiliaries.

5. Premodifying Stance Adverbs

The least frequently used stance marker in the L corpus was *premodifying stance adverb (stance adverb + adjective or noun phrase,* with only 6 occurrences. The following example is taken from one of the abstracts.

l) Virginia Woolf's *The Waves* is a difficult novel to comprehend rationally, and yet it is also a very moving novel that can take the reader from ecstasy to despair.

In this example, *very* is used to express the stance of the writer towards the novel.

## 5 Discussion

This study sought to explore the stance-marking grammatical devices academic writers use in two disciplines: Applied Linguistics and Literature. Two questions led the analysis of the collected data. The first asked about the way writers in the two areas express their stance, and the second about differences in the use of stance markers between the two disciplines. The results revealed that academic writers generally use a wide range of grammatical markers. The quantitative analysis presented above showed that the writers used most stance markers in Biber et al.’s (1999) framework and that writers often vary in their use of these markers. In doing so, writers create avenues for themselves to participate in the creation of the discourse, and more generally in shaping the world. In this respect, Strauss and Feiz (2014, p. 4) write:

*Stance-taking* is an inevitable consequence of participating in and producing discourse, of putting the world into words. Stance emerges in a speaker's or writer's choice of one linguistic form over another …. In all of these instances of discourse (and others), a speaker's or writer's stance is enacted and created; it is negotiated and re-negotiated. (italics in original)

In relation to the second research question, the results revealed that there was a similarity in the extensive use of stance complement clauses. This finding is similar to that of Jiang's and Hyland's (2015) study which found that noun complements are frequently used in soft fields such as applied linguistics and literature. However, the results showed that there were noticeable differences. First, hedging was only found in the AL corpus. This was also strengthened by a greater use of modals and semi-modals to express stance. This is attributed to the nature of the field of applied linguistics which is often described as ‘slippery’ in that it is characterised with uncertainty and ambiguity. In this respect, Davies (2007) points to the uncertainty of the field and argues that the “absence of certainty is much bemoaned by those who practise applied linguistics” (p. 1). This by no means alludes that literacy works are more scientific and that uncertainties do not exist; indeed, the two fields share some uncertainties in disseminating knowledge but writing in the literature discipline is more subjective and open for innovation and creativity which makes the use of boosting rather than hedging common among academic writers. However, literary research is more introspective than applied linguistics research.

It is also obvious that stance adverbials in the AL corpus were limited to two types only: Single adverbs and adverb phrases and hedges. However, all five types of stance adverbials were spotted in the L corpus. Specifically, prepositional phrases, adverbial clauses and comment clauses were only found in the L corpus. This may be attributed to the nature of literary studies which very often comes in the form of appropriation of a work in the various contexts. This warrants more references to how others appropriate the same work. In addition, writing in applied linguistics is generally of empirical nature which necessitates a typical generic structure (see Swales 1990). This structure makes it difficult for writers to use comment clauses in the abstract. The referencing style that is used in both areas is different—APA is used in applied linguistics research while MLA is the norm in literature research. This implies that—following the APA (see, for example, the *Publication Manual of the* *American Psychological Association* 7^th^ edition)—reference to other sources is not encouraged in the abstract. Finally, although the two journals from which the abstracts were taken are published by the same publisher—Oxford University Press—there are different instructions for how writers should produce their abstracts. These factors may have caused this obvious variegation in the way stance markers are used in the two sets of data. These differences strengthen the conventional view that—as Wette (2021, p. 106; italics in original) argues—although “there is a *common culture* of shared attributes across all academic subject areas”, there are views to the opposite; that is, there exist fundamental differences across disciplines in academic writing and other types of writing.

## 6 Conclusion

This study has tackled cross-disciplinary academic writing in terms of stance. In particular, it ventured to explore stance making in research article abstracts in the two areas of applied linguistics and literature. The results have demonstrated that although the examined areas are relatively related, writers in each discipline rely on different grammatical devices. These findings have implications for academic writing and EAP courses. First, EAP courses need to incorporate a wide range of disciplinary samples in order to apprise students of the differences in academic writing domains. Future research may venture to explore stance marking in other sections of the research article. Possible investigations might look into how stance is expressed throughout research articles. In addition, more cross-disciplinary studies are needed to enrich our understandings of the variegated means of expressing stance in academic writing. Cross-generic research shall also feed into the general understanding of stance making in writing courses in order to establish connections with other domains such as writing in the media. Finally, cross-cultural studies on the expression of stance in academic writing—particularly between English and Arabic—are rare and in need for more research. Similar to Wang (2006), for example, who conducted a study on how writers of newspaper commentaries in Chinese and English express stance, future studies can examine the expression of stance in English and Arabic. Such explorations may provide more insights to expand our knowledge and experience in writing for specific purposes, a need that is called for in mainstream literature about writing.

## Declarations

### Author contribution statement

Sharif Alghazo: Conceived and designed the experiments; Performed the experiments; Wrote the paper.

Mohd Nour Al Salem: Analyzed ​and interpreted the data; Wrote the paper.

Imran Alrashdan: Contributed reagents, materials, analysis tools or data; Wrote the paper.

Ghaleb Rabab'ah: Analyzed ​and interpreted the data; Wrote the paper.

### Funding statement

This research did not receive any specific grant from funding agencies in the public, commercial, or not-for-profit sectors.

### Data availability statement

The authors do not have permission to share data.

### Declaration of interests statement

The authors declare no conflict of interest.

### Additional information

No additional information is available for this paper.
